# Brain abscess of odontogenic origin: A case report and literature review

**DOI:** 10.1097/MD.0000000000036248

**Published:** 2023-12-01

**Authors:** Jingyi Wei, Feiyang Zhong, Lei Sun, Cheng-Yi Huang

**Affiliations:** a Center of Orthodontics, Department of Dentistry, Sir Run Run Shaw Hospital, Zhejiang University School of Medicine, Hangzhou, Zhejiang, China; b Department of Radiology, Sir Run Run Shaw Hospital, Zhejiang University School of Medicine, Hangzhou, Zhejiang, China.

**Keywords:** brain abscess of odontogenic origin, case report, metagenomic next-generation sequencing

## Abstract

**Background:**

The objective of this study is to investigate and understand the characteristics of odontogenic brain abscess.

**Methods:**

A case of brain abscess suspected to be caused by odontogenic infection was documented, and a comprehensive analysis and summary of odontogenic brain abscess cases reported in various countries over the past 20 years was conducted.

**Results:**

Based on the analysis and synthesis of both the present and previous reports, we have examined and consolidated the distinctive features of odontogenic brain abscess, the potential transmission pathway of pathogenic bacteria, diagnostic assertions, verification techniques, and crucial considerations during treatment.

**Conclusion:**

This investigation contributes to an enhanced comprehension and improved clinical identification of odontogenic brain abscess.

## 1. Introduction

Brain abscess refers to a localized purulent infection within the brain, which can be life-threatening in severe cases.^[1]^ Infection sources encompass various factors, including underlying diseases such as bacterial endocarditis or HIV infection, as well as conditions like organ transplantation, low immunity due to immunosuppressive drug treatment, maxillofacial trauma, or damage to the brain’s protective barrier following neurosurgery. Infections can also arise from otitis media, sinus or oral infections, or as secondary infections to other lesions. Notably, the etiology remains unidentified in 10% to 20% of cases.^[2,3]^ Among these cases, odontogenic and maxillofacial infections are relatively uncommon.^[4]^ Previous studies have documented the occurrence of brain abscesses resulting from chronic periodontitis, periapical periodontitis, dental caries, and other related conditions, as well as those caused by dental interventions. This article aims to present a case wherein bacteria of odontogenic origin were identified in the cerebrospinal fluid of a patient with a brain abscess, alongside the identification of severe periodontitis.

## 2. Case report

### 2.1. History of disease introduction

**Figure 1.**
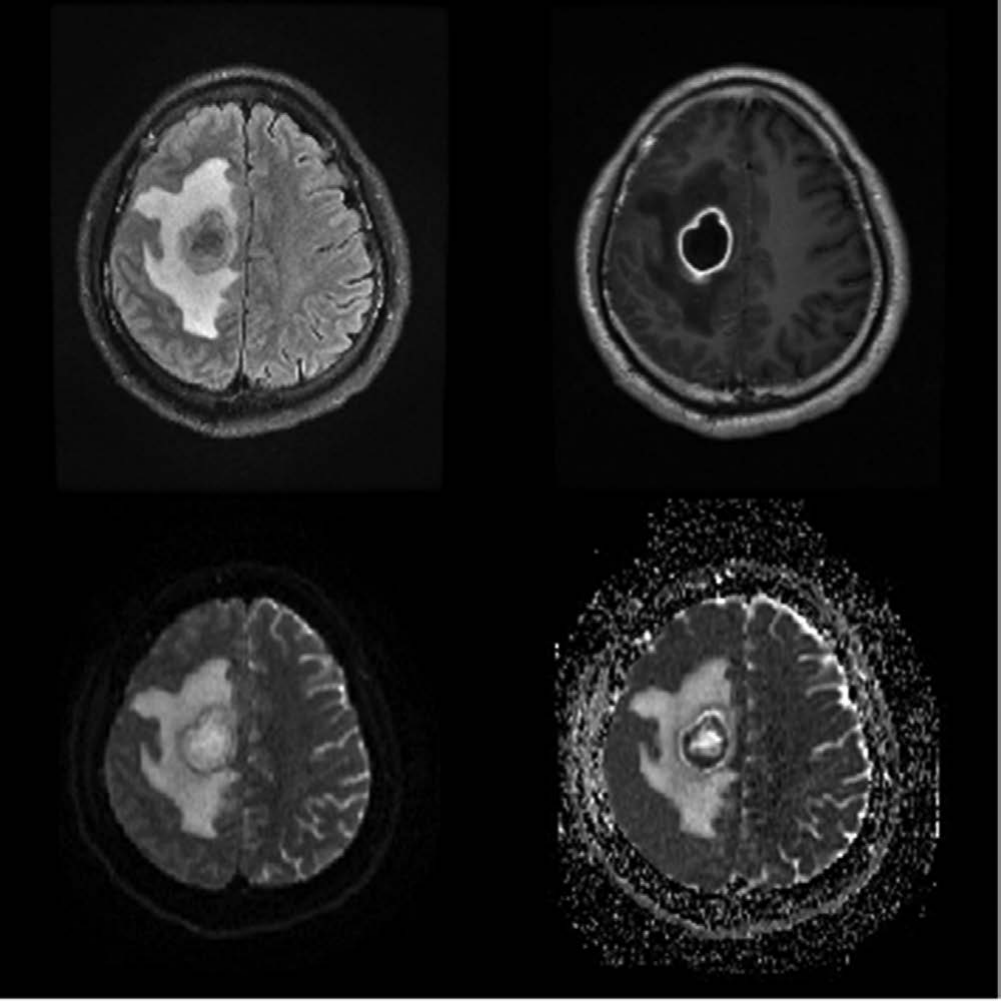
Imaging examination at admission.

The patient is a 43-year-old male. He was admitted to the Neurosurgery Department of Sir Run Run Shaw Hospital School of Medicine Zhejiang University (our hospital) due to fever, headache, and weakness of the left limb for 18 days. 18 days before admission, he was infected with COVID-19, and then had a low-grade fever. Two days later, he developed a headache with left limb weakness, unconsciousness, no nausea and vomiting, no limb convulsions, etc., so he went to the local people’s hospital for treatment. Considering intracranial infection, after anti-infection treatment, the cerebrospinal fluid of the lumbar puncture showed purulent changes (white blood cells 8000+, N96%, protein 3900+, glucose 0.13, chloride 124), anaerobic bacteria were found in metagenomic next-generation sequencing (mNGS) of cerebrospinal fluid: there were 1165 Porphyromonas endodontics and 96 Prevotella intermedia. The anti-infection was adjusted to meropenem and vancomycin, and the cerebrospinal fluid improved significantly after reexamination. After de-escalation treatment with antibiotics, the muscle strength of the right upper and lower limbs decreased significantly (grade 0–1). A plain head MR scan showed multiple lesions in the right frontal lobe and corpus callosum. The infectious lesions might be larger than the previous lesions, and the surrounding edema increased. For further diagnosis and treatment, he came to our hospital for neurosurgery. See (Fig. 1) for the space-occupying lesions in the imaging examination on admission. The laboratory examination showed purulent changes, and the detection of periodontal-derived bacteria in the external cerebrospinal fluid NGS. After questioning the medical history, the patient had a history of dental caries and periodontitis. He had cleaned his teeth in a local hospital half a year ago, but had never extracted them. Then he came to our department for further examination and treatment.

**Figure 2.**
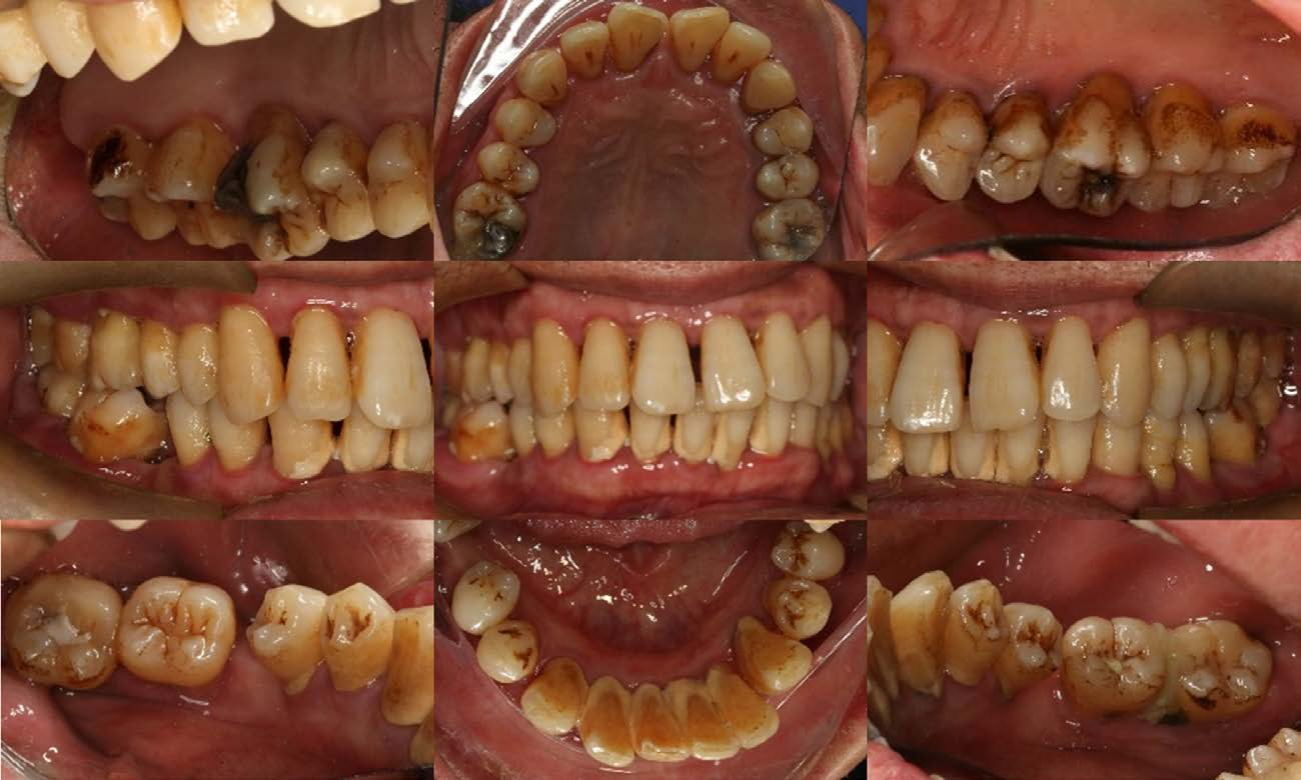
Intraoral pictures at the initial visit.

**Figure 3.**
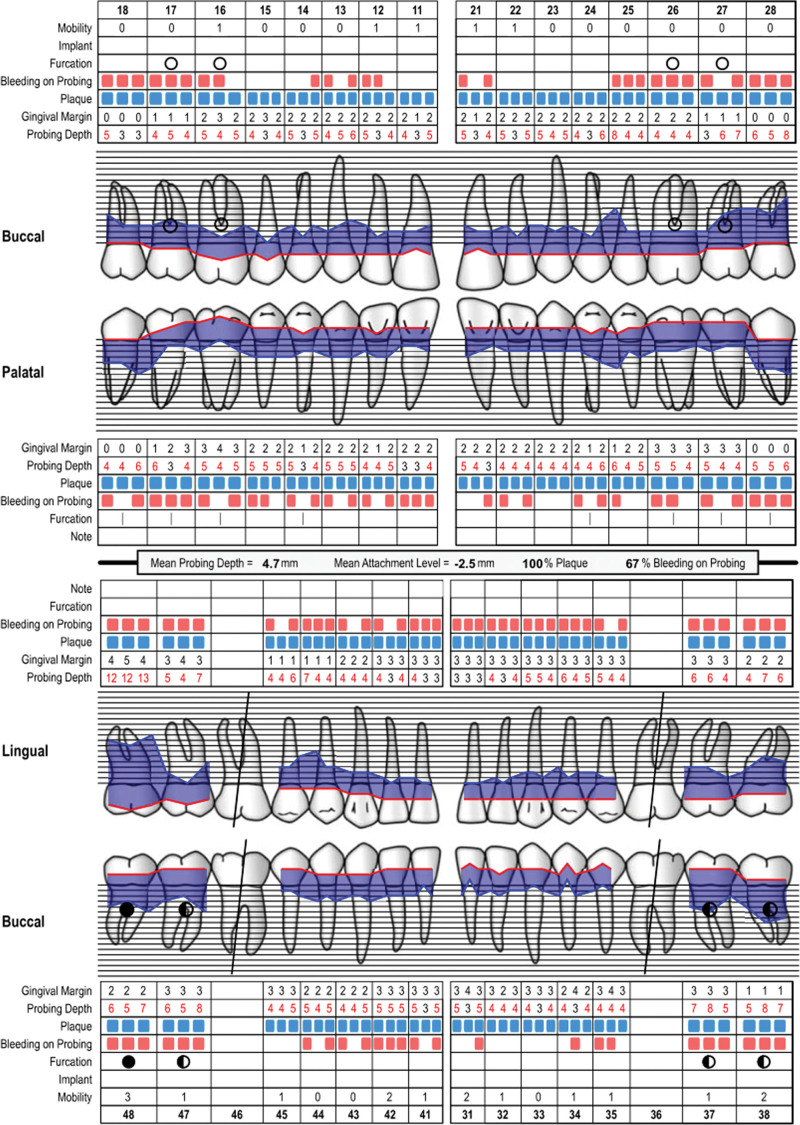
Periodontal chart at the initial visit.

**Figure 4.**
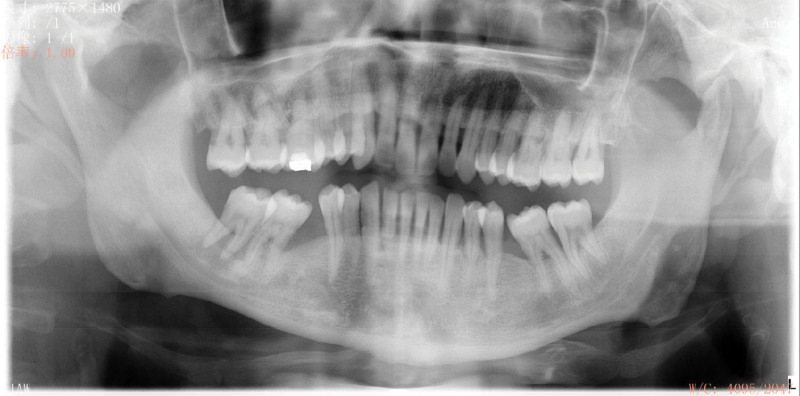
Panoramic radiograph at the initial visit.

**Figure 5.**
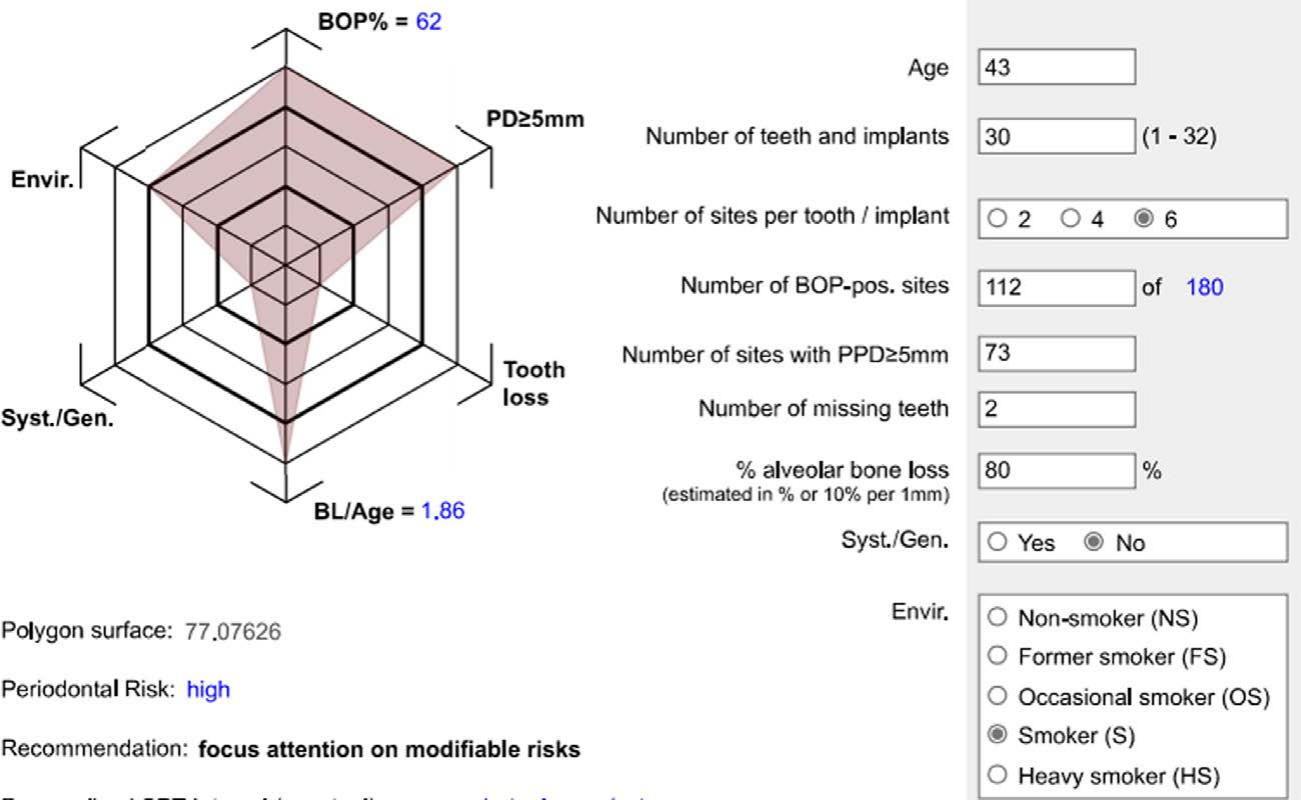
Periodontal disease risk assessment (PRA).

Oral clinical examination: the patient had poor oral hygiene. A large amount of debris and subgingival calculus were detected. The gingiva was red and swollen in full mouth. The teeth mobility of the full mouth was from I to III degree. For intraoral pictures, see (Fig. 2). Full mouth periodontal examination form (Fig. 3). A summary of the result of full mouth periodontal examination is given in Table 1. Radiographs showed(Fig. 4) horizontal alveolar bone loss around 48 was close to the apical area, and the horizontal alveolar bone loss around other teeth reached 1/3 to 2/3 of root length. The periapical radiolucent area was observed at 48. Make an individual periodontal risk assessment based on the model of Lang& Tonetti^[5]^ (Fig. 5).

**Table 1:** Summary of periodontal examination.

OHI-S	Plaque%	BOP%	PD	% of PD ≥ 4mm	GR	TM	FI
2	100%	62%	3–13 mm	80%	0–3 mm	0-III°	0–2°

BOP = bleeding on probing, FI = furcation index, GR = gingival recession, PD = probing depth, TM = tooth mobility

### 2.2. Preliminary diagnosis and treatment plan

Neurosurgery diagnosis: brain abscess (anaerobe infection)

Dental diagnosis: Periodontitis (stage III, grade C, according to the new classification of periodontitis^[6]^), 48 Chronic apical periodontitis.

Treatment plan: Neurosurgery gives treatment plan empiric anti-infection, dehydration, and other detumescence treatment, supplemented by neurotrophic drugs. Surgical treatment if necessary. At the same time, the periodontal treatment plan: periodontal system treatment, including oral hygiene education, full mouth ultrasonic supragingival and subgingival scaling, scaling, and root planing. The prognosis of 48 was poor, and extraction was recommended. Considering that the neutrophils during hospitalization were lower than 1*10^9/L, extraction would easily affect wound healing and infection, so extraction was postponed.

### 2.3. Treatment process

The neurosurgery department gave symptomatic and supportive treatment such as vancomycin, meropenem plus metronidazole empiric anti-infection, dehydration, and detumescence with mannitol, and combined with the rehabilitation department for rehabilitation training.

While the neurosurgery department continues to fight against infection, when the indicators were stable, the periodontal initial treatment, ultrasonic supragingival cleaning and ultrasonic subgingival cleaning would be started on February 2, 2023. After removing large supragingival calculus, the subgingival plaque was scraped for bacterial culture to verify the source of periodontal bacteria. Anaerobic bacteria were not effectively cultured. On February 12, 2023, subgingival plaque and granulation tissue from the deep periodontal pocket were examined by mNGS, and the pathogenic bacteria of periodontitis, Forsythia Forsythia, were found.

The patient’s condition improved significantly after guiding medication for anaerobic bacteria, and no neurosurgery was performed. The patient was discharged after 25 days and continued antibiotic treatment in a lower-level hospital. Advise the patient to continue periodontal treatment after the brain abscess subsides. The patient received anti-infection treatment in the local hospital for 3 months. The condition had improved but not fully recovered. Therefore, periodontal treatment will continue in the future.

## 3. Discussion

The prevalence of brain abscess is estimated to be 1 case per 100,000 individuals, with mortality rates ranging from 0 to 24 percent.^[1]^ With the improvement of medical detection technology and treatment methods, the mortality rate gradually dropped from 50% in the 1970s to 10%.^[7–9]^ Chronic periodontitis is a common disease in adults. Severe periodontitis is the sixth most prevalent disease in the world.^[10]^ About 10% of the world’s population is plagued by severe periodontitis.^[11]^ According to a retrospective study, about 13.6% of brain abscess infections originated from odontogenic etiology.^[12]^

Periodontitis is closely related to systemic diseases.^[13]^ Various evidences have shown that periodontitis is associated with cardiovascular disease, diabetes, adverse pregnancy outcomes, respiratory and digestive system diseases, and rheumatoid arthritis (RA). *Porphyromonas gingivalis (P. gingivalis*), *Tannerella forsythia*, and *Aggregatibacter actinomycetemcomitans* are well-documented pathogens of periodontitis.^[14]^ Laboratory periodontitis has been shown to induce inflammation in the brain.^[15]^ A review of the literature indicated that in immunocompetent patients, the most common pathogens of brain abscesses were streptococci (54%) and staphylococci (15%), followed by anaerobes.^[2,16]^

Due to the limited guidelines and reviews on odontogenic brain abscess, most of them are presented as case reports. The case reports of odontogenic brain abscess that we retrieved in the past 20 years are summarized in Table 2. And try to summarize some characteristics.

**Table 2:** Recently reported cases of brain abscess of odontogenic origin.

Authors	Year	Age, y, Sex	BA location	Oral diagnosis	Predisposing factors/ History	Microorganisms	Detection method
Marques, D.S.R., et al^[18]^	2004	60 female	Frontotemporal lobe	Periodontitis	Diabetes and previous head trauma	Streptococcus constellatus	Anaerobic culture followed by biochemical/enzymatic tests, ribotyping, and RAPD analyses were obtained from the brain abscess samples and periodontal pockets.
Antunes, A.A., et al^[17]^	2011	56, male	Temporal lobe	Periodontitis, caries, pulpitis	Not mentioned	Streptococcus viridans, Actinobacillus actinomycetemcomitans, Staphylococcus species	Bacterial culture of drained pus
Azenha, M.R, et al^[27]^	2012	70 male	Frontal lobe and occipital lobe	Caries, chronic apical periodontitis, periodontitis	Not mentioned	Streptococcus viridians and Bacteroides	Bacterial culture of drained pus
Rae Yoo, J, et al	2016	54, male	Parietooccipital lobe	Periodontitis	Recurrent episodes of toothache that was never treated.	*Porphyromonas gingivalis*.	Bacterial culture of drained pus
Akashi, M., et al^[28]^	2017	64, male	Temporal lobe	Advanced periodontitis	Clipping of a cerebral aneurysm 4 months before	Staphylococcus aureus	Bacterial culture of drained pus
Akashi, M., et al^[28]^	2017	68, male	Left lobe	Advanced periodontitis	Not mentioned	Streptococcus constellatus, Fusobac-terium nucleatum, Parvimonas micra	Bacterial culture of drained pus
Akashi, M., et al^[28]^	2017	64 female	Right lobe	Advanced periodontitis	Ventricular septal defect and Eisenmenger syndrome	Lactobacillus cate-naformis, *Porphyromonas gingivalis*, and F. nucleatum	Bacterial culture of drained pus
Viviano, M, et al^[20]^	2018	28, male	Parietal lobe and the occipital lobe	Periodontitis	Scaling and root planing	Streptococcus intermedius and Actinomyces.	Microbiological culture of drained pus and saliva.
Lajolo, C, et al^[21]^	2019	5, boy	Frontal and occipital lobes	multiple residual root	Not mentioned	No abnormal findings	Detected in the cerebrospinal fluid
Lin, J.H., et al^[26]^	2019	60, male	Anterior temporal lobe	A history of adenoid cystic carcinoma	Maxillectomy	Streptococcus Prevotella.	Metagenomic analyses
Costa, M.L, et al^[19]^	2020	7, boy	Fronto-parietal lobe	Acute apical periodontal abscess	Treatment for caries	Streptococcus intermedius	Bacterial culture of drained pus
Ribeiro, B, et al^[29]^	2021	66, female	Temporal and frontal lobes	Not mentioned	Dental procedure 1 month before	*Porphyromonas gingivalis*	Biopsy
Steiner C, et al^[30]^	2021	62 male	Frontal lobe	Peri-implantitis with sinus floor infection	Not mentioned	Streptococcus intermedius	Bacterial culture of drained pus
Ma, Z., et al^[31]^	2021	75 male	Temporal lobe	Not mentioned	Tooth extraction 1 month prior	Prevotella denticola and Fusobacterium nucleatum.	Metagenomics next-generation sequencing in the cerebrospinal fluid

From the point of view of the incidence population, male patients are in the majority. From the perspective of age distribution, adults are more likely to occur in the elderly aged 50 to 70, and a small number of cases occur in children aged 4 to 7. It is speculated that the awareness of oral hygiene in these 2 age groups is poor, the ability to maintain oral hygiene is not good, the systemic immunity is not as good as that of young and middle-aged people, the progress of oral infection is fast, the subjective symptoms are not obvious, and it is easy to cause other systemic diseases.

The infection routes of odontogenic brain abscess include direct contact infection of adjacent lesions, blood infection, etc.^[2,17]^ Sometimes it occurs under certain inducements, such as Marques, D.S.R. et al,^[18]^ because of diabetes and head trauma. The odontogenic brain abscess reported by Costa, M.L, et al in a 7-year-old child may be due to acute periapical abscess after dental caries treatment 3 weeks ago.^[19]^ Viviano M et al reported a rare brain abscess after professional periodontal treatment of a patent foramen ovale.^[20]^ Lajolo C. et al summarized the 3 most important predisposing factors for children’s brain abscess: immunosuppression, diabetes, and congenital heart disease.^[21]^ The patient in this case had severe periodontitis. Brushing teeth, flicking teeth, and chewing hard objects due to severe periodontitis may cause transient bacteremia.^[22]^ Furthermore, the patient has just experienced COVID-19, weakening his immunity. The possible way is that periodontal bacteria or their metabolites enter the blood and cause bacteremia, leading to brain abscess.

Regarding the detection method, CT and MRI can assist in checking the size, location, and changes of the abscess. Still, identifying the pathogenic bacteria of the brain abscess is of great significance to the treatment. Several papers have shown that.^[23,24]^ For infections of unknown origin, mNGS has expanded the bacterial detection spectrum and is a vital detection method. It is widely used in the early diagnosis and precise treatment of clinical infectious diseases. Compared with the traditional bacterial culture method, its detection time is shorter and more accurate in terms of oral cavity. Conducive to the early detection and treatment of diseases. Especially for anaerobic bacteria, the bacterial culture method is more difficult, and there is a risk of missed detection. There are about 774 species of bacteria in the mouth,^[25]^ and anaerobic bacteria are mainly prevalent in deep periodontal pockets.^[26]^ The diagnostic decisions of Lin, J.H. et al suggest that metagenomic sequencing should be considered for culture-negative samples.^[27]^ In previous case reports, bacteria were mainly detected from drained pus. Most of the detected bacteria are aerobic or facultative anaerobic bacteria such as streptococcus and staphylococcus, and a few are anaerobic bacteria. Sampling in periodontal pockets to prove the source of infection was not considered. We sampled anaerobic bacteria from deep periodontal pockets and cultured them, but no anaerobic bacteria were cultured. Then mNGS was used to detect bacteria in deep periodontal pockets. Although the same species of anaerobic bacteria were not detected in CSF, periodontitis-associated bacteria were detected. When the patient came to the Department of Stomatology, he was continuously infused with vancomycin and metronidazole for about 2 weeks. It was speculated that the concentration of anaerobic bacteria was extremely low.

Some previous literature discussed^[28]^that diagnosis of odontogenic brain abscess was difficult. Diagnostic features of odontogenic brain abscess proposed by Jeong Rae Yoo et al^[4,9,21]^: (1) no infection from other sources is found; (2) representative oral flora can be detected; (3) there must be clinical or radiological manifestations of periodontal disease. In this case, no potential source of infection in other parts of the patient was identified. The most direct and strong evidence was the discovery of odontogenic bacteria by metagenomic next-generation sequencing in cerebrospinal fluid, and the clinical and radiological manifestations of severe periodontitis.

In terms of treatment, management of a brain abscess includes local systemic infection control (antibiotic therapy with or without surgical drainage) and treatment of the primary lesion that caused the brain abscess. For the treatment of periodontitis, effective initial periodontal treatment can reduce the level of systemic inflammation. In order to avoid transient bacteremia from aggravating brain abscess, ultrasonic supragingival, and subgingival cleaning should be performed first. Root planning and other treatment should be performed after the infection was controlled and stabilized.

## 4. Summarize

Odontogenic brain abscesses are infrequent occurrences, and currently, there are no dental strategies specifically addressing brain abscesses. However, timely identification and intervention can significantly improve prognosis. This underscores the importance of preventive measures over curative approaches in clinical practice, prompting stomatologists to be vigilant regarding the correlation between patients and systemic diseases. Such attentiveness facilitates early detection and treatment of other rare ailments. Additionally, when patients from other medical disciplines are admitted for physical examinations, it is advisable for them to prioritize oral hygiene. In cases of infections with unidentified origins, early consideration of odontogenic sources may prove beneficial.

When encountering infections of unknown origin, they can consider early diagnosis of metagenomic next-generation sequencing, which is helpful for earlier targeted antibiotic treatment. For those that are clearly of oral origin, previous case reports have described a number of brain abscesses caused by oral treatment, suggesting that periodontists should do a good job in preoperative evaluation and preventive use of antibiotics when necessary when doing initial treatment for patients with poor immunity, Gentle operation, to avoid transient bacteremia caused or aggravated brain abscess. Strengthen daily oral hygiene guidance to patients after treatment, and the prevention and treatment of periodontitis can help reduce the risk of brain abscess.

## Acknowledgments

Special thanks to all participants who took part in this study.

## Author contributions

**Conceptualization:** Feiyang Zhong.

**Formal analysis:** Lei Sun.

**Methodology:** Jingyi Wei.

**Software:** Jingyi Wei.

**Writing – original draft:** Jingyi Wei, Feiyang Zhong, Cheng-Yi Huang.

**Writing – review & editing:** Jingyi Wei, Feiyang Zhong, Cheng-Yi Huang.
